# The influence of the NOD *Nss1*/*Idd5 *loci on sialadenitis and gene expression in salivary glands of congenic mice

**DOI:** 10.1186/ar2300

**Published:** 2007-09-27

**Authors:** Trond Ove R Hjelmervik, Anna-Karin Lindqvist, Kjell Petersen, Martina Johannesson, Anne-Kristin Stavrum, Åsa Johansson, Roland Jonsson, Rikard Holmdahl, Anne Isine Bolstad

**Affiliations:** 1 Department of Oral Sciences-Periodontology, Faculty of Dentistry, University of Bergen, Årstadveien, N-5009 Bergen, Norway; 2 Broegelmann Research Laboratory, The Gade Institute, University of Bergen, Haukelandsveien, N-5021 Bergen, Norway; 3 Cartela AB, Scheelevägen, SE-220 07 Lund, Sweden; 4 Computational Biology Unit, Bergen Center of Computational Biology, University of Bergen, Høyteknologisenteret, Thormøhlensgate, N-5008 Bergen, Norway; 5 Psychiatric Genetics, The Wellcome Trust Centre for Human Genetics, University of Oxford, Roosevelt Drive, Oxford OX3 7BN, UK; 6 Department of Clinical Medicine, University of Bergen, Haukeland University Hospital, Jonas Lies vei, N-5020 Bergen, Norway; 7 Medical Inflammation Research, University of Lund, Sölvegatan, 221 84 Lund, Sweden

## Abstract

The nonobese diabetic (NOD) *Nss1 *and *Idd5 *loci have been associated with sialadenitis development in mice. In this study the NOD *Nss1 *and *Idd5 *loci were backcrossed onto the healthy control strain B10.Q by using the speed congenic breeding strategy, resulting in three congenic strains: B10.Q.*Nss1*, B10.Q.*Nss1*/*Idd5 *heterozygous and B10.Q.*Nss1*/*Idd5 *homozygous. We investigated the effects of the *Nss1 *and *Idd5 *loci on sialadenitis and gene expression in NOD congenic mice. One submandibular salivary gland from each mouse was used for histological analysis of sialadenitis, whereas the contralateral salivary gland was used for gene expression profiling with the Applied Biosystems Mouse Genome Survey chip v.1.0. The results were validated using quantitative reverse transcriptase PCR. The NOD *Nss1 *and *Idd5 *loci had clear influence on the onset and progression of sialadenitis in congenic mice. Double congenic mice exhibited the most severe phenotype. We successfully identified several genes that are located in the NOD congenic regions to be differentially expressed between the congenic strains and the control strain. Several of these were found to be co-regulated, such as *Stat1*, complement component C1q genes and *Tlr12*. Also, a vast contingency of interferon-regulated genes (such as *Ltb*, *Irf7 *and *Irf8*) and cytokine and chemokine genes (such as *Ccr7 *and *Ccl19*) were differentially expressed between the congenic strains and the control strain. Over-representation of inflammatory signalling pathways was observed among the differentially expressed genes. We have found that the introgression of the NOD loci *Nss1 *and *Idd5 *on a healthy background caused sialadenitis in NOD congenic mouse strains, and we propose that genes within these loci are important factors in the pathogenesis. Furthermore, gene expression profiling has revealed several differentially expressed genes within and outside the NOD loci that are similar to genes found to be differentially expressed in patients with Sjögren's syndrome, and as such are interesting candidates for investigation to enhance our understanding of disease mechanisms and to develop future therapies.

## Introduction

Primary Sjögren's syndrome (pSS) is an autoimmune disease (AID) hallmarked by ocular and oral dryness, known as keratoconjunctivitis sicca and xerostomia, respectively. Lymphocytic infiltrates in the lacrimal and salivary glands (SGs) are prominent features. Sjögren's syndrome can occur alone or secondary to other autoimmune connective tissue diseases, such as rheumatoid arthritis and systemic lupus erythematosus [1].

pSS is considered a multifactorial disease, in which the onset and progression are invoked by environmental factors in genetically susceptible individuals. The genetic contribution to pSS by rates of monozygotic concordance in twins has not yet been studied, whereas the concordance rate for different types of AID is ranging from 15% to 60% [2]. Familial clustering of AID has frequently been reported, and it is common for a Sjögren's syndrome (SS) proband to have relatives with other AIDs [3,4]. There is substantial body of evidence supporting an association of SS with the major histocompatibility complex (MHC) class II region [5,6], but the association with formation of anti-Ro/La antibodies is stronger than that with the disease itself for the alleles DRB1*03 and DQB1*02 [6].

Studies conducted to identify polymorphisms in cytokine genes [7] and other candidate genes associated with SS [3] have been inconclusive. However, recent gene expression studies of minor SGs from SS patients have demonstrated several cytokine genes and interferon-regulated genes to be upregulated in SS patients compared with control individuals, indicating that these genes are important players in the pathology of SS [8-11].

There is a need to unravel the key mechanisms of onset and progression of multifactorial AIDs to enhance our understanding and to improve diagnostics and treatment. The search for underlying mechanisms can be facilitated by reducing the heterogeneity of environmental and genetic factors using murine models of the human condition. The nonobese diabetic (NOD) mouse, originally introduced to study type 1 diabetes [12], has been widely used as a model for AIDs. In addition to insulitis [12], this strain develops SS-like features such as reduced exocrine function and focal lymphocytic infiltrates in the SGs [13], and it has become a well established mouse model of SS [14].

By exchanging the NOD MHC class II allele *H2*^*g*7 ^with *H2*^*q*^, Johansson and coworkers [15] developed a NOD.Q strain that is protected from type 1 diabetes but exhibits the same incidence of sialadenitis as the NOD strain [15]. It was concluded that the genes responsible for sialadenitis development probably reside outside the MHC region. By linkage analysis of the (NOD.Q × B10.Q)F_2 _intercross, in which 9% of the F_2 _animals exhibited clear signs of sialadenitis, the *Nss1 *locus on chromosome 4 was found to be associated with sialadenitis development [15]. Previously, Brayer and coworkers [16] found the *Idd5 *locus on chromosome 1 to be linked to sialadenitis.

The speed congenic breeding strategy was developed to selectively breed specific loci on the genome and to reduce genetic heterogeneity between the strains of mice, so that the only genetic differences between the experimental strain and the control strain lie within the locus of interest [17]. In the present study the two NOD loci *Idd5 *and *Nss1 *were backcrossed onto the SS-resistant B10.Q background using the speed congenic breeding strategy. Three congenic strains were established: the *Nss1 *homozygous single congenic strain; the *Nss1 *homozygous and *Idd5 *heterozygous double congenic strain; and the *Nss1 *homozygous and *Idd5 *homozygous double congenic strain.

The purpose of the present study was to monitor the influence of each of these two loci on the incidence and severity of sialadenitis, and to identify genes located within the NOD loci that are differentially expressed in submandibular SGs from the three congenic strains compared with B10.Q (control strain). We conclude that the *Idd5 *and *Nss1 *loci residing outside the MHC are sufficient for development of sialadenitis. Furthermore, we found several genes residing within these two loci to be differentially expressed, and possibly are important factors in the pathogenesis.

## Materials and methods

### Mice

**Figure 1:**
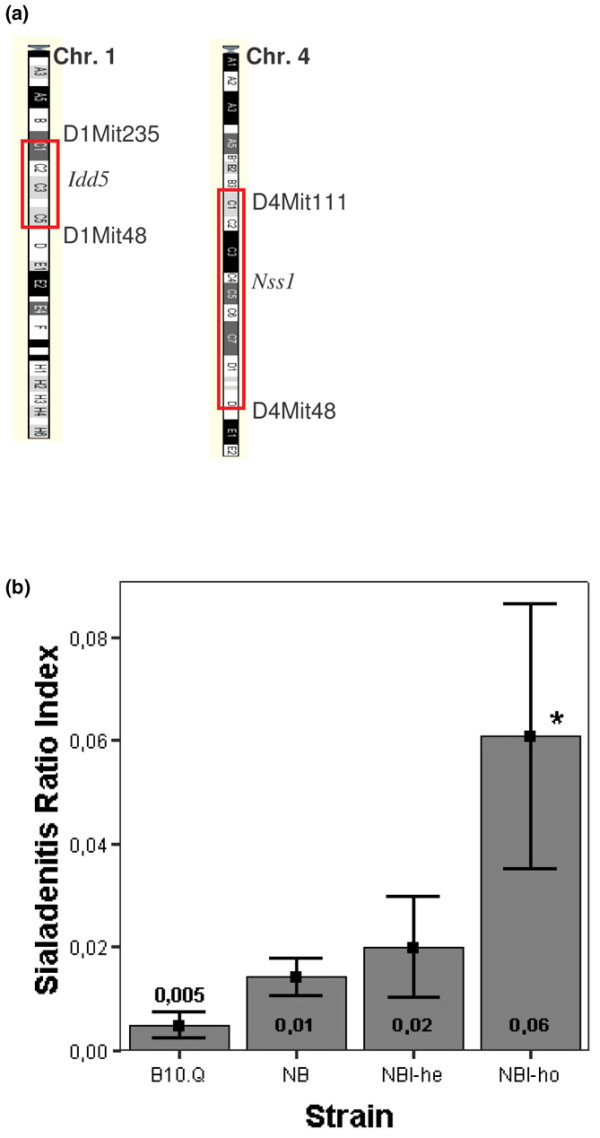
Congenic regions and incidence of sialadenitis in congenic mice and healthy controls. **(a) **The location of the two nonobese diabetic (NOD) fragments *Idd5 *and *Nss1 *on chromosome (Chr.) 1 and Chr. 4, respectively. **(b) **The severity of sialadenitis, represented by the Sialadenitis Ratio Index (SRI), which is the total focus area (mm^2^) divided by the total gland area (mm^2^). The strains are B10.Q (*n *= 10), B10.Q.*Nss1 *(NB; *n *= 4), B10.Q.*Nss1*/*Idd5*-he (NBI-he; *n *= 4) and B10.Q.*Nss1*/*Idd5*-ho (NBI-ho; *n *= 7). The numbers and each of the bars represent mean values of SRI for each group, the error bars show standard error of the mean, and statistically significant increase compared with B10.Q is indicated by asterisk (analysis of variance with Dunnet's *post hoc *test; *P *= 0.017).

Congenic strains in the present study are all bred on C57BL/10 (*H2*^*q*^) (B10.Q) background. The congenic strain for the *Nss1 *locus was established by backcrossing the *Nss1 *region on chromosome 4 [15] from NOD.Q onto B10.Q using the speed congenic technique [17] for eight generations and at the end intercrossed for two generations. Similarly, the *Idd5 *region on chromosome 1 [16] from the NOD.Q was backcrossed to the B10.Q background for seven generations and intercrossed. The double congenic mice were established by intercrossing seventh generation mice from *Nss1 *backcross-breeding and *Idd5 *breeding, respectively, to generate mice homozygous at *Nss1 *in combination with heterozygosity or homozygosity at *Idd5*. Genotyping was performed for each generation. Three congenic strains containing the loci of interest on a B10.Q background were established: B10.Q.*Nss1 *(homozygous NOD.Q *Nss1 *locus on a B10.Q background), B10.Q.*Nss1*/*Idd5*-he (homozygous NOD.Q at *Nss1*, heterozygous for NOD.Q derived *Idd5*) and B10.Q.*Nss1*/*Idd5*-ho (homozygous NOD.Q at *Nss1*, homozygous NOD.Q at *Idd5*). The NOD fragment *Idd5 *ranged from 46 to 89 megabases on chromosome 1 flanked by the markers D1Mit48 and D1Mit235, and *Nss1 *ranged from 53 to 140 megabases on chromosome 4 flanked by the markers D4Mit48 and D4Mit111, as shown in Figure 1a. The congenic strains were genotyped across the whole genome to ensure a pure B10.Q background.

The C57BL/10 (*H2*^*q*^) strain, B10.Q, originated from Professor Jan Klein (Tübingen, Germany) and was sister-brother maintained as the B10.Q/rhd strain. The *H2*^*q *^congenic NOD strain, NOD.Q, was developed as described previously [15] and maintained as NOD.Q/rhd.

The mice were bred, kept and used in animal facilities under standard conditions at the animal unit at Medical Inflammation Research, University of Lund, Lund, Sweden. The mice were maintained in a climate controlled environment with a 12-hour light/dark cycle in polystyrene cages containing wood shavings; they were fed with standard rodent chow and were given free access to water (as defined by Medical Inflammation Research [18]). All animal experiments were approved by the local ethics committee in Malmö-Lund.

### Evaluation of incidence and severity of sialadenitis

The mice were killed at age 16 to 19 weeks, and the pairs of submandibular SGs were removed. One SG from each mouse was frozen in RNAlater (Ambion, Austin, TX, USA) for subsequent gene expression analysis, whereas the other was embedded in TissueTek^® ^(Sakura Finetek USA, Inc., Torrance, CA, USA), snap frozen in liquid nitrogen and kept at -80°C until later haematoxylin and eosin staining. Tissue sections (6.0 μm thick) were prepared in a cryostat at -20°C. The sections were dried and fixed in 50% cold acetone (4°C) for 30 seconds, followed by 100% acetone (4°C) for 5 minutes. After air drying (1 minute), the slide was submerged in haematoxylin for 25 seconds, washed in water, submerged in eosin for 20 seconds, and washed in ethanol and cold toluene for 5 minutes. After short evaporation, cover glass was mounted with two drops of Eukitt (Kindler Gmbh & Co., Freiburg, Germany). The foci, defined as inflammatory mononuclear cell infiltrates with more than 50 mononuclear cells/mm^2 ^of the submandibular SG section [19], were identified and the severity of inflammation was determined by calculating the focus area relative to the total area of the gland [15]. The differences in inflammation between the congenic strains and the control strain were examined for statistical significance by using one-way analysis of variance with a two-tailed Dunnet's *post hoc *test at a significance level of 0.05.

### Preparation of total RNA

The SGs frozen in RNAlater were divided in two and homogenized, first manually in liquid nitrogen and thereafter in 350 μl RLT buffer from the RNeasy MiniKit (Qiagen Inc., Valencia, CA, USA) was added to the tissue powder and the sample was homogenized in a Kinematica Polytron homogenizer (Brinkman, Westbury, NY, USA) for 1 minute, and the RNeasy MiniKit was used for RNA purification (as described by the manufacturer). The RNA was eluted in 60 μl RNase free water and precipitated with white glycogen, as described by Hjelmervik and coworkers [8]. The RNA quality was examined on the 2100 Bioanalyzer (Agilent Technologies, Palo Alto, CA, USA). All samples included high amounts (from 2 to 5 μg/μl) of good quality RNA.

### Salivary gland gene expression

The total RNA was submitted to the RNA-in/data-out service of the Norwegian Microarray Consortium Core Facility in Bergen, Norway. The labeling of 1.0 μg total RNA for each individual was done according to the NanoAmp™ RT-IVT Labeling Kit protocol (Applied Biosystems [ABI], Foster City, CA, USA). The cDNA was purified and subjected to IVT labeling at 37°C for 9 hours. The digoxigenin (DIG)-labeled cRNA was purified using the columns supplied with the kit and eluted in 100 μl nuclease free water. The yield and quality of the labeled cRNA was measured at an absorbance of 260 nm.

The DIG labeled cRNA was fragmented, hybridized to ABI Mouse Genome Survey chip v.1.0 and scanned in accordance with the ABI Chemiluminescence detection kit protocol.

### Data processing and statistical analysis

The gene expression data was analyzed using J-Express Pro 2.7 (Molmine, Bergen, Norway [20]). All of the microarray procedure was MIAME (Minimum Information About a Microarray Experiment) compliant. The microarray data were deposited in the ArrayExpress repository with the accession number E-BASE-6 [21].

#### Pre-processing 1: filtration and normalization of microarray data

The data files from the ABI Mouse Genome Survey microarray v.1.0 were processed using J-Express Pro to filter and normalize the data from each hybridization and compile gene expression profile matrix (gene by sample) datasets for further analysis. The pre-normalized intensity values were extracted per spot (ASSAY_NORMALIZED_SIGNAL) from the data files, and all flagged, weak and control spots were filtered out (FLAGS > 1.0, S/N < 3.0, PROBE_TYPE != probe). Before they were compiled into an expression profile data matrix, all arrays were quantile normalized in order to be comparable. Genes with at most 15% missing values were allowed in the final dataset. The signal intensities in the dataset were further log transformed (base 2), and missing values were replaced by the average of nearest rows neighbour values. One individual, B10.Q.*Nss1*/*Idd5*-ho 11, was found to be an outlier in both the array plot and the correspondence analysis in J-Express, and was excluded from the raw dataset, before filtration and normalization. The resulting gene expression matrix was used in the 'standard analysis'.

#### Alternative analysis: selecting for genes with standard deviation >0.7

The raw data were processed with the same parameters as in pre-processing 1, except for the S_N > 3 filter. After log2 transforming the intensities, replacing missing values and collapsing probes to genes, the gene lists were filtered by minimum standard deviation = 0.7. Finally, the genes in the list 'pre-processing 1' were removed. The resulting gene expression matrix was used for the 'alternative analysis'.

#### Differentially expressed gene analysis

The search for differentially expressed genes was performed both on a single gene and gene set level. The significance analysis of microarrays (SAM) [22] implementation in J-Express was used to look for differentially expressed genes on a gene by gene basis, whereas gene set enrichment analysis (GSEA) [23] was used to look for sets of genes, sharing common characteristics, that were differentially expressed between the classes examined.

#### Compilation of gene sets

Gene sets were created using the Panther Biological Process and Panther Molecular Function, extracted from the Applied Biosystems Mouse Annotation File, dated 12 December 2005. The hierarchy of the Panther ontologies has at most three levels. We created one gene set for each of the biological processes and molecular functions, on all levels. The gene sets at the finest level contains only the genes annotated to this particular process or function, whereas a parent process or function contains all genes annotated to this level plus all the genes annotated to its children. All probes listed in the annotation file are annotated to all levels, which enabled us to create complete gene sets in a straight forward manner. This yielded 238 gene sets based on Panther Biological Processes and 251 gene sets based on Panther Molecular Functions.

#### Parameters of GSEA

Probes were collapsed to genes, using Primary Gene Id from the ABI Mouse Annotation File, before running GSEA. Gene sets smaller than five were excluded from the analysis. The default signal to noise ratio metric was used to rank the genes. Significance of the gene set analysis was tested by permuting class labels (1,000 iterations). Default values were also used for all other parameters.

### Pathway search

The lists of differentially expressed genes from the 'standard analysis' and 'alternative analysis' were combined for each comparison, and the 'Probe Id' for each gene was applied in the pathway search in the Panther Classification System [24]. The Mouse AB 1700 gene list, representing all the genes on the microarray, was used as reference gene list and Bonferroni correction for multiple testing was applied.

### Verification of the microarray results by quantitative reverse transcriptase PCR

The quantitative reverse transcriptase PCR (QPCR) was carried out in all individuals in each group, using the same extract of total RNA as used for the microarrays. The cDNA was synthesized in volumes of 50 μl according to the recommendations for the TaqMan Reverse Transcriptase kit (ABI). The reverse transcription of total RNA was carried out as described by Bolstad and colleagues [10]. The cDNA was kept at -20°C until use. Genes for verification of the microarray results by QPCR were selected from among the genes differentially expressed between congenic strains and control strain, from the gene lists of both standard and alternative analyses. Primers and probes were purchased as TaqMan^® ^Assays-on-Demand™ Gene Expression Products (ABI). These are pre-formulated assays (250 μl, 20× mix) containing two unlabelled PCR primers and one FAM™ dye-labelled TaqMan^® ^MGB probe each. The 10 assays used for QPCR validation were *Ccl19 *(Mm00839967_g1), *Cd19 *(Mm00515420_m1), *Egf *(Mm00438696_m1), *Klk9 *(*Egf-bp*) (Mm00658534_mH), *Ltb *(Mm00434774_g1), *Sell *(Mm00441291_m1), *Zap70 *(Mm00494255_m1), *Stat1 *(Mm00439518_m1), *Dock7 *(Mm01259863_m1) and *Fas *(Mm00433237_m1). The cDNA was mixed with the TaqMan^® ^Assays-on-Demand™ primers and probe in a 2× TaqMan Universal Master Mix (ABI) and run in 10 μl triplicate PCR in a 384-well tray (ABI). The endogenous controls *Gapdh*, β-*actin *and *18s *were run on all samples, and *Gapdh *was used for normalizing the total RNA added in each reaction. The ΔΔCt method was used to calculate relative mRNA level, and statistically significant differences were determined using the two tailed Student's *t*-test, at a significance level of 0.05. For each assay, one reaction without Multiscribe RT enzyme was included, to ensure RNA specificity of the assays.

## Results

### Influence of NOD *Idd5 *and *Nss1 *loci on severity of sialadenitis

Ten mice of the control strain B10.Q and mice from three congenic strains, namely B10.Q.*Nss1 *(*n *= 4), B10.Q.*Nss1*/*Idd5*-he (*n *= 4) and B10.Q.*Nss1*/*Idd5*-ho (*n *= 7), all of which were female, were killed at age 16 to 19 weeks, and the submandibular SGs were removed. All mice appeared healthy upon visual inspection.

Histological examination of the SGs demonstrated the severity of sialadenitis to be increased with NOD loci introduced to the B10.Q background, as shown in Figure 1b. The control mice had none or in some cases one minor focal infiltrate, whereas the number and the severity of infiltrates in the NOD congenic mice increased considerably. In order to assess the effect that addition of each NOD locus to the B10.Q background exerted on sialadenitis in the mice, the mean focus area over total gland area (termed Sialadenitis Ratio Index [SRI]), was calculated for each strain of congenic mice and compared with that of the control strain. The B10.Q.*Nss1 *strain (*n *= 4) and the B10.Q.*Nss1*/*Idd5*-he strain (*n *= 4) had an increased SRI compared with the control strain, although not statistically significantly so. The B10.Q.*Nss1*/*Idd5*-ho strain (*n *= 7) exhibited the most severe sialadenitis, and the SRI was found to be significantly increased compared with the B10.Q strain (*n *= 10) by analysis of variance with Dunnet's *post hoc *test (*P *= 0.017).

### Gene expression differences between the congenic mice and the control strain

The contralateral submandibular SG of each animal was used for gene expression profiling. Initially, we observed a high degree of similarity in the gene expression from the strains of mice heterozygous or homozygous for the *Idd5 *locus (there were no differentially expressed genes). The two strains B10.Q.*Nss1*/*Idd5*-he and B10.Q.*Nss1*/*Idd5*-ho were therefore grouped to enhance the power of the gene expression analysis. The double congenic mice as one group of mice were denoted B10.Q.*Nss1*/*Idd5*.

The analysis was focused mainly on differentially expressed genes from three comparisons: B10.Q versus B10.Q.*Nss1*, B10.Q versus B10.Q.*Nss1*/*Idd5*, and B10.Q.*Nss1 *versus B10.Q.*Nss1*/*Idd5*. The SAM algorithm was run on the comparisons and gene lists were created based on a false discovery rate (FDR) of less than 10% for the standard analysis gene list (genes with a signal to noise ratio >3 in all samples) and FDR less than 20% for the alternative analysis gene list (hypervariable genes with minimum standard deviation >0.7). In comparing the gene expression of the congenic strains with that of the control strains, the standard analysis elicited gene lists of 2,435 differentially expressed genes for B10.Q versus B10.Q.*Nss1*/*Idd5 *and 1,411 differentially expressed genes for B10.Q versus B10.Q.*Nss1 *and 255 and 78, respectively, differentially expressed genes from the alternative analysis. Therefore, 10% to 20% of the expressed genes (remaining after pre-processing the data) were differentially expressed between the congenic mice and the control mice. For B10.Q.*Nss1 *versus B10.Q.*Nss1*/*Idd5*, only 192 genes from the standard analysis and 51 genes from the alternative analysis were differentially expressed. Thus, 2% of the expressed genes were differentially expressed when comparing the single congenic with the double congenic mice.

### Quantitative PCR validation of gene expression data

Genes were selected from the gene lists of the standard and alternative analyses in order to verify the microarray results using QPCR. Only genes with FDR below 60% in at least one of the three gene lists were selected. Table 1 shows the genes selected for QPCR and displays the fold change from the microarray and QPCR as a mean of all individuals in the respective groups.

**Table 1:** Validation of microarray by QPCR

Gene symbol	B10.Q versus B10.Q.*Nss1*			B10.Q versus B10.Q.*Nss1*/*Idd5*			B10.Q.*Nss1 *versus B10.Q.*Nss1*/*Idd5*		
	
	Array FC	QPCR FC	QPCR *P *value	Array FC	QPCR FC	QPCR *P *value	Array FC	QPCR FC	QPCR *P *value
*Ltb*	2	2.4	0.03	2.7	4.3	0.01	1.3	1.8	0.25
*Egf*	-3	-5.9	0.04	-1.9	-1.6	0.17	1.6	3.5	0.01
*Ccl19*	2.7	2.5	< 0.01	3.3	8.6	0.01	1.2	3.5	0.17
*Klk9*	-2.2	-5.0	0.03	-1.4	-1.3	0.44	1.5	3.8	< 0.01
*Stat1*	2.2	1.3	0.3	1.8	1.6	0.03	-1.3	1.3	0.28
*Zap70*	2.8	2.5	< 0.01	2.4	3.6	0.02	1.1	1.5	0.34
*Cd19*	3.1	5.4	0.02	3.5	20.9	0.04	1.2	3.9	0.25
*Sell*	-	-	-	3	9.2	0.09	2.7	3.7	0.25
*Dock7*	-1.6	-1.9	< 0.01	-1.8	-1.6	< 0.01	-1.1	1.2	0.2
*Fas*	2.3	1.2	0.34	2	1.5	0.01	-	-	-

The three columns under each comparison (gene list) represent, from left to right, the fold change (FC) from the microarray gene list, the FC for the quantitative reverse transcriptase PCR (QPCR) and the statistical significance from the QPCR (*P *value). Genes with positive FC were upregulated in the congenic mice compared with B10.Q, and upregulated in B10.Q.*Nss1*/*Idd5 *when compared with B10.Q.*Nss1*. The genes *Ltb*, *Egf*, *Ccl19*, *Klk9 *and *Stat1 *were selected from the gene list of the standard analysis, and *Zap70*, *Cd19*, *Sell*, *Dock7 *and *Fas *were selected from the gene list of the alternative analysis.

All gene expression quantifications obtained in the QPCR were in accordance with results from the microarray. The B10.Q versus B10.Q.*Nss1 *gene list was verified by seven genes, four that were upregulated and three that were downregulated in B10.Q.*Nss1*, at a significance level of 0.05. Furthermore, two genes were found upregulated by QPCR. The B10.Q versus B10.Q.*Nss1*/*Idd5 *gene list was verified by seven genes, six of which were upregulated in B10.Q.*Nss1*/*Idd5*. Furthermore, two genes were found to be downregulated and one upregulated. For B10.Q.*Nss1 *versus B10.Q.*Nss1*/*Idd5*, two genes verified the microarray data, both of which were upregulated in B10.Q.*Nss1*/*Idd5*; these genes were the only two with FDR below 60% for this gene list. Additionally, five of the genes were upregulated as on the microarray.

### Gene graph analysis and K-means clustering

**Figure 2:**
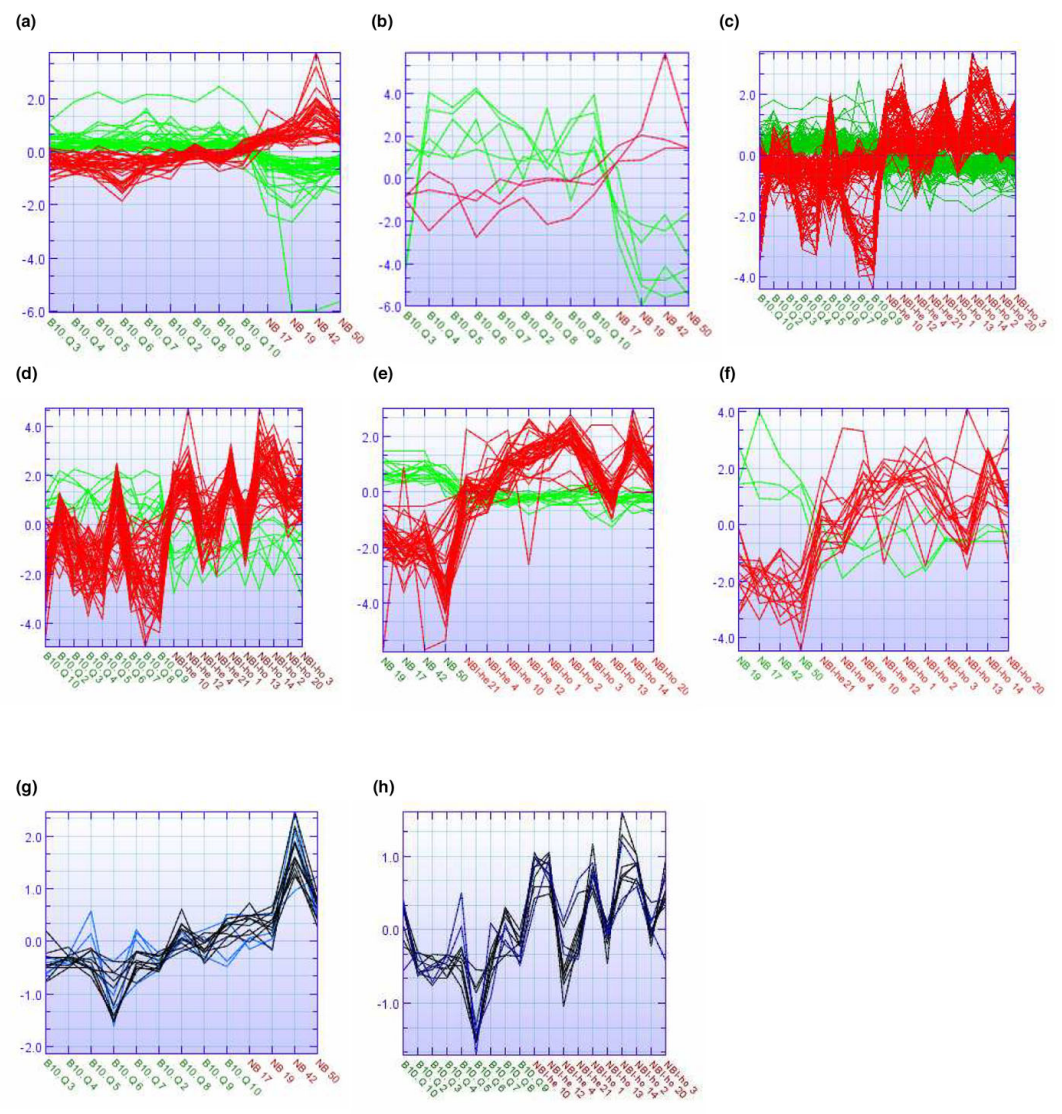
Genes differentially expressed in congenic mice compared to healthy controls. Panels a to f show gene graphs of genes differentially expressed with false discovery rate of 0.0: **(a) **B10.Q versus NB, 'standard analysis' (71 genes); **(b) **B10.Q versus NB, 'alternative analysis' (8 genes); **(c) **B10.Q versus NBI, 'standard analysis' (271 genes); **(d) **B10.Q versus NBI, 'alternative analysis' (61 genes); **(e) **NB versus NBI, 'standard analysis' (59 genes); and **(f) **NB versus NBI, 'alternative analysis' (19 genes). Upregulated and downregulated genes in the congenic mice are presented by red and green lines, respectively. The genes within the nonobese diabetic (NOD) congenic fragments on the lists of differentially expressed genes were selected and clustered by K-means clustering to identify co-regulation of genes within the fragments. Also presented are differentially expressed genes in fragments *Idd5 *(blue) and *Nss1 *(black) for the **(g) **B10.Q versus NB and **(h) **B10.Q versus NBI comparisons. The gene expression values are log2 transformed and mean normalized. The abscissa indicates the individual mice and the ordinate shows the gene expression intensities. The congenic mice are denoted NB (B10.Q.*Nss1*) and NBI (B10.Q.*Nss1*/*Idd5*-he and B10.Q.*Nss1*/*Idd5*-ho).

Although the congenic mice varied genetically from the control strain at only one or two NOD loci, there was a substantial difference in gene expression. This was convincingly demonstrated in that the top differentially expressed genes with a FDR of 0.0 (no false positive in the gene list) had distinct gene profiles that distinguished the control mice from the congenic mice. These gene graphs are presented in Figure 2a–f for both the standard and alternative analyses. The number of genes with no FDR and consistent expression within the groups illustrate that the dataset is robust, and that the NOD loci are able to induce a consistent influence on the gene expression. Among the top differentially expressed genes (FDR = 0.0), there was an even distribution of upregulated and downregulated genes. This clustering shows that the gene expression data are robust and provide clear differences between the strains.

### Gene expression differences of genes located in the NOD loci *Idd5 *and *Nss1*

The main focus of the gene expression profiling has been on identifying genes in the two NOD derived congenic regions *Idd5 *and *Nss1 *that clearly cause the increase in sialadenitis in the congenic mice compared with the control strain. Differentially expressed genes within the congenic regions for each of the three gene list are presented in Table 2.

**Table 2:** Differentially expressed genes in the two congenic intervals

Gene ID	Gene symbol^a^	Gene name	Fold change^b^		
			
			B10.Q versus NB	B10.Q versus NBI	NB versus NBI
215627	*Zbtb8*	Zinc finger and BTB domain containing 8	3.7	4.4	
384059	*Tlr12*	Toll-like receptor 12	3.3		
381549	*Zfp69*	Zinc finger protein 69	3.2	3.3	
230779	*Serinc2*	Serine incorporator 2	3	2.2	
16818	*Lck*	Lymphocyte protein tyrosine kinase	2.9	3	
12260	*C1qb*	Complement component 1, q subcomponent, beta polypeptide	2.8	1.8	
23833	*Cd52*	CD52 antigen	2.5	2	
12259	*C1qa*	Complement component 1, q subcomponent, alpha polypeptide	2.4	1.7	
20846	*Stat1*	Signal transducer and activator of transcription 1	2.2	1.8	
14268	*Fn1*	Fibronectin 1	2.2	1.7	
14612	*Gja4*	Gap junction membrane channel protein alpha 4		2.2	
18991	*Pou3f1*	POU domain, class 3, transcription factor 1		2.2	
11520	*Adfp*	Adipose differentiation related protein	2.1	1.7	
107581	*Col16a1*	Procollagen, type XVI, alpha 1	2.1	1.6	
269582	*Clspn*	Claspin homolog (Xenopus laevis)		2.1	
16792	*Laptm5*	Lysosomal-associated protein transmembrane 5	1.9	1.8	
12579	*Cdkn2b*	Cyclin-dependent kinase inhibitor 2B (p15, inhibits CDK4)	1.9	1.6	
20970	*Sdc3*	Syndecan 3	1.9	1.6	
20720	*Serpine2*	Serine (or cysteine) peptidase inhibitor, clade E, member 2	1.8		
14700	*Gng10*	Guanine nucleotide binding protein (G protein), gamma 10		1.8	
230657	*Tmem69*	Transmembrane protein 69	1.7	1.5	
16008	*Igfbp2*	Insulin-like growth factor binding protein 2	1.7		
73723	*Sh3bgrl3*	SH3 domain binding glutamic acid-rich protein-like 3	1.7		
93691	*Klf7*	Kruppel-like factor 7 (ubiquitous)	1.6		-1.6
66290	*Atp6v1g1*	ATPase, H+ transporting, V1 subunit G isoform 1	1.6		
27981	*D4Wsu53e*	DNA segment, Chr 4, Wayne State University 53, expressed	1.6		
17965	*Nbl1*	Neuroblastoma, suppression of tumorigenicity 1	1.6		
67103	*Ltb4dh*	Leukotriene B4 12-hydroxydehydrogenase			1.6
230379	*Asah3l*	N-acylsphingosine amidohydrolase 3-like			1.6
19231	*Ptma*	Prothymosin alpha	1.5		
68777	*Tmem53*	Transmembrane protein 53		1.5	
320438	*Alg6*	Asparagine-linked glycosylation 6 homolog (yeast, alpha-1,3,-glucosyltransferase)		1.5	
101739	*Psip1*	PC4 and SFRS1 interacting protein 1			1.5
230861	*Eif4g3*	Eukaryotic translation initiation factor 4 gamma, 3	-1.5	-1.7	
74648	*S100pbp*	S100P binding protein	-1.5		
101739	*Psip1*	PC4 and SFRS1 interacting protein 1	-1.5		
18710	*Pik3r3*	Phosphatidylinositol 3 kinase, regulatory subunit, polypeptide 3 (p55)	-1.5		
67694	*Ift74*	Intraflagellar transport 74 homolog (Chlamydomonas)	-1.5		
76850	*Eif2c4*	Eukaryotic translation initiation factor 2C, 4		-1.5	
19359	*Rad23b*	RAD23b homolog (S. cerevisiae)		-1.5	-1.8
319146	*Ifnz*	Interferon zeta			-1.5
100206	*Adprhl2*	ADP-ribosylhydrolase like 2		-1.6	-1.7
230815	*Man1c1*	Mannosidase, alpha, class 1C, member 1		-1.6	
67299	*Dock7*	Dedicator of cytokinesis 7	-1.6	-1.8	
21885	*Tle1*	Transducin-like enhancer of split 1, homolog of Drosophila E(spl)	-1.7		
66902	*Mtap*	Methylthioadenosine phosphorylase		-1.8	
11363	*Acadl*	Acetyl-coenzyme A dehydrogenase, long-chain			-1.8
71872	*Aox4*	Aldehyde oxidase 4	-1.9		
236511	*Eif2c1*	Eukaryotic translation initiation factor 2C, 1	-2.1	-1.8	
58864	*Tssk3*	Testis-specific serine kinase 3	-3.2		
13370	*Dio1*	Deiodinase, iodothyronine, type I	-3.4	-2.3	
66264	*Ccdc28b*	Coiled coil domain containing 28B	-4.2	-4.3	
66260	*Tmem54*	Transmembrane protein 54	-5	-8	
74754	*Dhcr24*	24-Dehydrocholesterol reductase	-12.7	-13.8	

^a^Genes on chromosome 1 (*Idd5*) lie within the markers D1Mit48 and D1Mit235, and genes on chromosome 4 (*Nss1*) lie within the markers D4Mit48 and D4Mit111. Genes with underlined gene symbols are in the *Idd5 *fragment. Unknown expressed sequence tags and genes with fold change < ± 1.5 were removed. ^b^The fold change is the relative intensities for each gene from the microarray gene lists B10.Q versus B10.Q.*Nss1*, B10.Q versus B10.Q.*Nss1*/*Idd5 *and B10.Q.*Nss1 *versus B10.Q.*Nss1*/*Idd5*, respectively. Genes with positive fold change were upregulated in the congenic mice, and in B10.Q.*Nss1*/*Idd5 *when compared with B10.Q.*Nss1*. NB, B10.Q.*Nss1*; NBI, B10.Q.*Nss1*/*Idd5*-he and B10.Q.*Nss1*/*Idd5*-ho.

Usually, one will find that the expression of some genes located in the same chromosomal regions and contributing in the same process is co-regulated. In order to identify genes in the *Idd5 *and *Nss1 *loci that are co-regulated, the differentially expressed genes from (within) the two loci were subjected to K-means clustering. Thus, 91 genes from the comparison B10.Q versus B10.Q.*Nss1 *and 131 genes from the comparison B10.Qversus B10.Q.*Nss1*/*Idd5 *were clustered into five clusters for each comparison. We identified a set of genes that clustered together in both comparisons (Figure 2g,h), namely *Stat1*, *Fn1*, *Cd52*, *Col16a1*, *Laptm5 *and *Sdc3*, and the complement component 1 genes *C1qb*, *C1qa *and *C1qg*.

Located in the *Nss1 *locus, *Lck *and *Tlr12 *were among the most highly upregulated genes in B10.Q.*Nss1*/*Idd5 *compared with B10.Q, and *Ifnz *was downregulated in B10.Q.*Nss1*/*Idd5 *compared with B10.Q.*Nss1*.

Furthermore, from Table 2 it can be concluded that a majority of the genes from the *Nss1 *region are common between the gene lists (34 out of 47 genes), whereas only two out of eight genes in the *Idd5 *region are common. This can be explained by that the *Nss1 *region in both congenic strains was similarly NOD derived, whereas the *Idd5 *region differed between the single and double congenic strains, and we therefore saw more diverse gene expression from this region.

Among the most highly differentially expressed genes, we found 24-dehydrocholesterol reductase (*Dhcr24*), transmembrane protein 54 (*Tmem54*), coiled coil domain containing 28B (*Ccdc28b*) and testis-specific serine kinase 3 (*Tssk3*) all to be downregulated. Furthermore, we found zink finger and BTB domain containing 8 (*Zbtb8*) and zink finger protein 69 (*Zfp69*) genes to be among the most highly upregulated in the congenic mice.

### Differentially expressed immunity related genes

In breeding the congenic strains, great care was taken only to transfer the designated NOD fragments *Idd5 *and *Nss1 *to the B10.Q background. However, passenger genes may travel with the NOD fragments. Additionally, the genes within the NOD fragments may affect gene expression elsewhere in the genome. We therefore looked for differentially expressed genes outside the NOD fragments, especially genes related to inflammation.

In the B10.Q versus B10.Q.*Nss1 *gene list of 1,411 differentially expressed genes, 20 interferon-regulated genes, including *Ifnγ*, and 16 out of 16 MHC genes (classes I and II) were all upregulated in B10.Q.*Nss1*. The *Tlr2, Tlr5 *and *Tlr12 *genes (encoding Toll-like receptors 2, 5 and 12), as well as several complement component 1 genes, were upregulated in B10.Q.*Nss1*.

The B10.Q vs B10.Q.*Nss1*/*Idd5 *comparison also exhibited several interferon-regulated genes, of which 29 out of 30 were upregulated and 18 out of 18 MHC genes were upregulated. *Tlr2 *was upregulated in B10.Q.*Nss1*/*Idd5*. In both B10.Q.*Nss1 *and B10.Q.*Nss1*/*Idd5*, the gene encoding Toll-interleukin1 receptor domain containing adapter (*Tirap1*) was downregulated compared with B10.Q.

Several other cytokines and chemokines were upregulated in the congenic mice compared with the control strain, as listed in Table 3. In B10.Q.*Nss1*/*Idd5*, 18 chemokines and seven cytokines were differentially expressed, and in B10.Q.*Nss1 *13 chemokines and eight cytokines were upregulated. Several chemokines, among these lymphotoxin β (*Ltb*), *Cxcl13*, *Cxcl16 *and *Ccl19*, and the cytokines *Socs1 *and *Socs3 *were commonly upregulated in both B10.Q versus B10.Q.*Nss1 *and B10.Q versus B10.Q.*Nss1*/*Idd5 *gene lists. There were no cytokines or chemokines identified as being differentially expressed between the two congenic strains B10.Q.*Nss1 *and B10.*Q.Nss1*/*Idd5*.

**Table 3:** Differentially expressed cytokine and chemokine genes

Gene ID	Gene symbol	Gene name	Fold change^a^	
			
			B10.Q versus NB	B10.Q versus NBI
55985	*Cxcl13*	Chemokine (C-X-C motif) ligand 13	4.3	4.8
17329	*Cxcl9*	Chemokine (C-X-C motif) ligand 9	4.2	3.1
20307	*Ccl8*	Chemokine (C-C motif) ligand 8	3.6	3
20304	*Ccl5*	Chemokine (C-C motif) ligand 5	3	2.3
20305	*Ccl6*	Chemokine (C-C motif) ligand 6	2.9	2
24047	*Ccl19*	Chemokine (C-C motif) ligand 19	2.7	3.3
12772	*Ccr2*	Chemokine (C-C motif) receptor 2	2.5	1.7
12766	*Cxcr3*	Chemokine (C-X-C motif) receptor 3	2.3	2.2
66102	*Cxcl16*	Chemokine (C-X-C motif) ligand 16	2.3	1.5
12702	*Socs3*	Suppressor of cytokine signalling 3	2.1	2
450136	*Ltb*	Lymphotoxin B	2	2.7
20308	*Ccl9*	Chemokine (C-C motif) ligand 9	2	1.5
12703	*Socs1*	Suppressor of cytokine signalling 1	2	1.6
12774	*Ccr5*	Chemokine (C-C motif) receptor 5	2	1.5
75974	*Dock11*	Dedicator of cytokinesis 11	1.8	1.8
67299	*Dock7*	Dedicator of cytokinesis 7	-1.6	-1.8
109006	*Ciapin1*	Cytokine induced apoptosis inhibitor 1	-1.5	-1.3
20315	*Cxcl12*	Chemokine (C-X-C motif) ligand 12	1.4	1.4
57266	*Cxcl14*	Chemokine (C-X-C motif) ligand 14	1.4	1.2
20292	*Ccl11*	Small chemokine (C-C motif) ligand 11	1.3	1.4
54199	*Ccrl2*	Chemokine (C-C motif) receptor-like 2	1.3	1.2
56066	*Cxcl11*	Chemokine (C-X-C motif) ligand 11		2.4
12775	*Ccr7*	Chemokine (C-C motif) receptor 7		2.3
12767	*Cxcr4*	Chemokine (C-X-C motif) receptor 4		2

^a^The fold change is the relative intensities for each gene from the microarray gene lists B10.Q versus B10.Q.*Nss1 *and B10.Q versus B10.Q.*Nss1*/*Idd5*. Genes with positive fold change were upregulated in the congenic mice. Genes with fold change <1.3 were removed. NB, B10.Q.*Nss1*; NBI, B10.Q.*Nss1*/*Idd5*-he and B10.Q.*Nss1*/*Idd5*-ho.

### Gene set enrichment analysis

Thus far the gene expression analysis has been based on gene lists with differentially expressed single genes (where the focus is often on the most highly differentially expressed genes or on genes implicated beforehand). In order to look for more subtle gene expression changes than the highest ranking differentially expressed genes, and to identify molecular and biological processes where several genes are involved in the same process, gene sets were generated and the GSEA was carried out.

GSEA was based on gene lists from the four comparisons (B10.Q versus B10.Q.*Nss1*, B10.Q versus B10.Q.*Nss1*/*Idd5*, B10.Q.*Nss1 *versus B10.Q.*Nss1*/*Idd5 *and B10.Q.*Nss1*/*Idd5*-he versus B10.Q.*Nss1*/*Idd5*-ho), with all of the genes remaining after the 'signal to noise' filtration in 'pre-processing 1'. Gene sets with a FDR below 25%, corresponding to each normalized enrichment score [23], are considered significantly enriched in the gene lists.

From the 'Biological processes' branch of the Gene Ontology, the gene set 'Other carbon metabolism' was significantly enriched in B10.Q.*Nss1*/*Idd5*-he compared with B10.Q.*Nss1*/*Idd5*-ho, and several carbonic anhydrase genes contributed positively to the enrichment score.

Several gene sets were enriched with a level of significance of 1% (GSEA nominal *P *value) in B10.Q.*Nss1 *and B10.Q.*Nss1*/*Idd5 *compared with B10.Q, such as 'Chemokine mediated immunity', 'Macrophage mediated immunity', 'T-cell and B-cell mediated immunity' and 'Immunity and defense'. This trend was further heavily supported by the fact that 10 out of the 12 gene sets (out of 238) related to 'Immunity' were among the top 14 ranking gene sets in the B10.Q versus B10.Q.*Nss1*/*Idd5 *comparison.

### Pathway search

In order to have any impact on the biological process in the SG, several genes in the same signalling pathway are usually expressed simultaneously. If several genes in our gene list belong to the same pathway, then this pathway is likely to be important for the biological processes that take place in the SG, and therefore these genes may be amenable for further investigation as candidates for therapeutic intervention.

**Figure 3:**
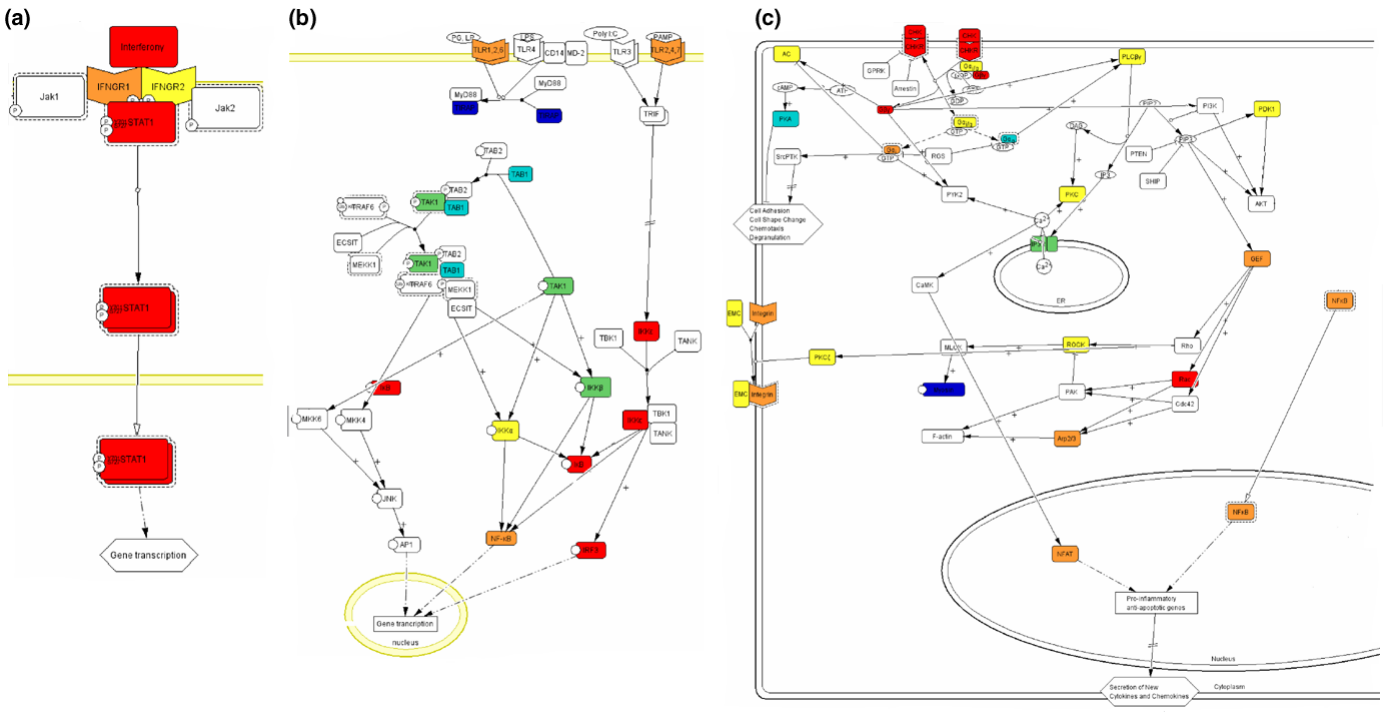
Pathways over-represented in the lists of differentially expressed genes. **(a) **'Interferon gamma signalling pathway' in the B10.Q versus B10.Q.*Nss1 *gene list; **(b) **'Toll-like receptor signalling pathway'; and **(c) **'Inflammation mediated by chemokine and cytokine signalling pathway' in the B10.Q versus B10.Q.*Nss1*/*Idd5 *gene list. The coloured boxes represent the genes present in our gene list, and the colours indicate fold change (FC), where positive FC indicates upregulation in the congenic mice. Red and blue genes have FC above 2 and FC below -2, respectively. Orange and green have FC from 1.5 to 2 and -2 to -1.5, respectively. Yellow and light blue are upregulated and downregulated, respectively, with FC < 1.5.

The lists with differentially expressed genes resulting from gene expression profiling were therefore submitted to a Panther database search in order to identify signalling pathways over-represented in the gene lists. Three pathways were over-represented in both the B10.Q versus B10.Q.*Nss1 *and the B10.Q versus B10.Q.*Nss1*/*Idd5 *gene lists: 'T-cell activation', 'Inflammation mediated by cytokine and chemokine signalling pathway', and 'B-cell activation'. In addition, the 'Interferon-gamma signalling pathway' was over-represented in B10.Q versus B10.Q.*Nss1*. Genes in the 'Toll-like receptor signalling pathway' were over-represented, but not significantly so at a significance level of 0.05 after Bonferroni correction for multiple testing (in this case 131 signalling pathways). Parts of three pathways are displayed in Figure 3.

## Discussion

This study was conducted in order to monitor the effect of the two NOD fragments *Idd5 *and *Nss1 *on sialadenitis development and to identify genes in the two loci associated with the development of sialadenitis in the congenic mice.

All the congenic strains had higher SRI values than the control strain, although not all of these were significant. The NOD congenic mouse strain homozygous in both *Nss1 *and *Idd5 *loci (NBI-ho) had the most severe sialadenitis. The introgression of only the NOD-*Nss1 *fragment to a healthy control genotype indicated that this was sufficient for spontaneous development of sialadenitis. Because of the low sample number in this group (a result of difficulties with the breeding of the congenic animals) the increase was not significant. However, this trend was supported by observations reported by Johansson and coworkers [15] in an F_2 _intercross, in which only the *Nss1 *locus was associated with sialadenitis and not the *Idd5 *locus.

To reproduce the *Idd5 *locus, as indicated by Brayer and coworkers [16], we made double congenics in which the *Idd5 *congenic fragment was introgressed on the B10.Q.*Nss1 *strain. In accordance with previous findings [16], we expected to see enhanced sialadenitis in mice with the homozygosity but not heterozygosity of *Idd5*. However, our findings indicated an increase in severity of sialadenitis also in the strain that was heterozygous for the *Idd5 *fragment, which might be explained by dosage dependent increase in SRI values with the addition of the *Idd5 *locus. As expected, the most severe phenotype was seen in the double congenic mice, with both *Nss1 *and *Idd5 *congenic fragments present homozygously. This implies that the genes within the two fragments may work synergistically for the development of sialadenitis, and that expression of possible candidate genes is influenced by the presence of both fragments.

The congenic breeding strategy enabled us to focus on the genes within the limits of the NOD loci, which evidently were causative for the sialadenitis, and to pinpoint the main factors in the pathogenesis by differential gene expression. We thus identified several genes within the NOD loci that were differentially expressed in the congenic animals compared with the healthy controls, and potential candidates that may play a role in the pathogenesis (Table 2).

In previous studies dissecting AIDs by combining congenic strains and microarray technology for type 1 diabetes [25] and collagen-induced arthritis [26], only few differentially expressed genes have been identified. In our comparison of the congenic strains with the control strain, as many as 20% of the expressed genes were identified as being differentially expressed, and several of these were located in the congenic fragments. This may be because we used larger congenic fragments and whole genome microarrays. On the contrary, when comparing the two congenic strains with each other, we found that only about 2% of the expressed genes were differentially expressed. This was expected because the two congenic strains only differed in the *Idd5 *locus. However, the results from the gene expression data are robust, and the gene graphs illustrate that we were able to identify clear differences between the strains in each comparison.

The mechanism for onset of SS is as yet unknown, although it has been hypothesized that exogenous agents such as virus can precede the vicious circle of chronic inflammation, causing an induction of type 1 interferons as a defence mechanism [27]. The expression of type 1 interferon-regulated genes has been found to be increased in SGs from SS patients in previous studies [8,9]. Båve and coworkers [11] identified increased numbers of interferon-α producing cells in SG biopsies from SS patients. The present study demonstrates upregulation of gene expression for *Ifnγ *and several interferon-induced genes and transcription factors, including *Stat1*, and *Irf1*, *Irf7*, *Irf2 *and *Irf8 *in the NOD congenic mice. A study conducted by Cha and colleagues [28] illustrated an important role for *Ifnγ *in exocrinopathy, in which neither the NOD *Ifnγ *null nor the NOD *IfnγR *(encoding interferon-γ receptor) null mice developed sialadenitis or secretory dysfunction, like their NOD littermates did.

Furthermore, interferon-γ is known to induce *Ltb *signalling and expression of chemokines, and to induce the Jak/STAT (Janus kinase/signal transducer and activator of transcription) signalling pathway by upregulating *Stat1 *[29], as we reported in this study. We found *Socs1 *and *Socs3 *to be upregulated, although suppressor of cytokine signalling (SOCS)1 and SOCS3 are negative regulators of the Jak/STAT signalling pathway [29,30]. *Socs *genes were found to be upregulated in other autoimmune inflammatory diseases, such as *Socs3 *in T cells from the colon mucosa of patients with Crohn's disease [31] and over-expression of SOCS1, SOCS2 and SOCS3 in the epidermis of psoriatic skin lesions, as well as *in vitro *stimulation with interferon-γ induced *Socs1 *and *Socs3 *expression in keratinocytes [32].

That interferons can induce chemokine expression was reflected in our data. We found numerous chemokines to be upregulated in the congenic mice (Table 3), such as *Ltb*, B-cell activating *Cxcl13 *and its receptor RANTES (*Cxcr5*), and T-cell activating *Cxcl12 *[7,33], which is consistent with our previous findings in SS patients [8]. This facilitates the formation of high endothelial venules and enhances the ability of lymphocytes to permeate through the endothelial lining and into the SG [33]. The reduced gene expression of *Ifnz *is of interest because the gene lies in the *Nss1 *congenic region. The gene product is known to be inhibitory of B-cell development [34], and the downregulation of this gene in the double congenic mice may be related to progression of B-cell development in the target organ. Monoclonal B-cell proliferation and lymphoma development is a common finding in the SGs of pSS patients [35,36].

We also found up-regulation of the genes encoding the CC chemokine receptor (CCR)7 and its ligand CC chemokine ligand (CCL)19. The ligands CCL19 and CCL21, and their receptor CCR7 have been found to be involved in homing of lymphocytes to the target organ [37] and to be localized in the lymphocytic infiltrates of the synovium in patients with rheumatoid arthritis [38]. Studies have been conducted in which *Ccr7 *deficient mice develop sialadenitis [39]. This is explained by the lack of central tolerance established in this mouse model. A most interesting finding reported by Ju and coworkers [40] indicated upregulation of *IRF8 *gene expression in dendritic cells by transforming growth factor-β_1_, which subsequently induced CCR7 gene expression. In our study both *Irf8 *and the chemokine receptor CCR7 gene, present on T-cells and dendritic cells, were found to be upregulated in the congenic mice; this makes sense, based on the reported presence of lymphocytic infiltrates in the SG [41].

We found several complement component C1q genes and *Tlr12*, located in the congenic fragments, to be upregulated in the congenic mice. Stimulation of Toll-like receptors induces type I interferon production, and plays an important role in SS [9]. In a recent study, the complement component C1q in serum from patients with systemic lupus erythematosus was found to be upregulated, and further upregulated in patients with active disease [42].

The inflammation is thought to result in SG tissue destruction and dysfunction in pSS. Therefore, apoptosis has been a much studied area in pSS research. Studies of apoptosis in SGs from SS patients have demonstrated increased expression of the apoptosis inducing Fas and Fas ligand genes [10], whereas the degree of apoptosis was reduced in the focal infiltrates and unchanged in the glandular epithelial cells [43,44]. The balance between pro-apoptotic *Bax *and anti-apoptotic *Bcl2 *was found to be crucial for the induction of apoptosis [43].

In the present study *Ifnγ*, *Fas *and *Bcl2a1 *were found to be upregulated in the NOD congenic mice, and this is in accordance with previous findings on apoptosis in pSS patients [44]. Caspases are fundamental molecules in any apoptotic pathway. We found several caspases in our gene lists (*Casp1 *and *Casp11 *were upregulated, and *Casp7 *was slightly downregulated). *Casp3 *and *Casp6*, which are known to inherit the effector function [45], were not found to be differentially expressed.

From the Panther search, we found pathway terms for 'Inflammation mediated by chemokine and cytokine signalling'. This pathway precedes the induction of cytokine and chemokine gene expression, which is reflected by the large number of proinflammatory cytokines and chemokines found to be differentially expressed in the congenic mice.

We were unable to detect any gene expression differences in the comparison of the two congenic strains heterozygous and homozygous for *Idd5 *locus by regular SAM analysis, although this had much influence on the severity of sialadenitis. However, by GSEA we identified a set of genes belonging to 'Carbon metabolism' that was enriched between the strains. This gene set contains several carbonic anhydrase genes that were downregulated in B10.Q.*Nss1*/*Idd5*-ho compared with B10.Q.*Nss1*/*Idd5*-he. Downregulation of carbonic anhydrases has also been found associated with pSS [8,46]. The GSEA found three transcription/translation related gene sets enriched in B10.Q compared with B10.Q.*Nss1*, and one ribosome related gene set enriched in B10.Q.*Nss1 *compared with B10.Q. Several gene sets related to immune reactions were enriched in the congenic mice, although not significantly so. This method is powerful for achieving high throughput and unbiased categorization of the gene lists into functional relationships. However, the method is dependent on the number of samples in each group [23], which may have been a limiting factor for utilizing the full potential of GSEA in the present study.

## Conclusion

We set out to identify genes within two regions outside the MHC region previously identified by quantitative trait locus mapping as being involved in the development of murine sialadenitis [15,16]. First, we developed a congenic mouse model that differed genotypically from the control mice only in two non-MHC loci, namely *Nss1 *and *Idd5*. The two loci together were sufficient for the congenic mice to develop sialadenitis spontaneously. Furthermore, we have shown that the loci affect the gene expression profiles in the SGs of the congenic mice. The gene expression reflected the congenic fragments. When comparing the gene expression of the B10.Q.*Nss1 *and the B10.Q.*Nss1*/*Idd5 *congenic strains with the control strain, several genes within the fragments were identified as being differentially expressed, especially cytokines, chemokines and other immunity-related genes.

## Abbreviations

AID = autoimmune disease; CCL = CC chemokine ligand; CCR = CC chemokine receptor; FDR = false discovery rate; MHC = major histocompatibility complex; NOD = nonobese diabetic; PCR = polymerase chain reaction; pSS = primary Sjögren's syndrome; QPCR = quantitative reverse transcriptase PCR; SAM = significance analysis of microarrays; SG = salivary gland; SOCS = suppressor of cytokine signalling; SRI = Sialadenitis Ratio Index; SS = Sjögren's syndrome.

## Competing interests

The authors declare that they have no competing interests.

## Authors' contributions

All authors participated in the writing of the manuscript. TORH and AIB participated in the design of the study, collection of organs, data collection and data analysis. AKL, RH, AJ and MJ participated in the breeding of the mice and collected the organs. KP and AKS participated in the microarray data analysis. AKL, RH and RJ participated in the design of the study.
